# *GIDInd*: an automated indexing software for grazing-incidence X-ray diffraction data

**DOI:** 10.1107/S1600576721006609

**Published:** 2021-07-30

**Authors:** Manuel Peter Kainz, Lukas Legenstein, Valentin Holzer, Sebastian Hofer, Martin Kaltenegger, Roland Resel, Josef Simbrunner

**Affiliations:** a Institute of Solid State Physics, Graz University of Technology, Petersgasse 16, Graz, 8010, Austria; b Division of Neuroradiology, Vascular and Interventional Radiology, Medical University Graz, Auenbruggerplatz 9, Graz, 8036, Austria

**Keywords:** grazing-incidence X-ray diffraction, indexing, crystallographic unit cells, software

## Abstract

Grazing-incidence X-ray diffraction (GIXD) is a widely used technique for the crystallographic characterization of thin films. The identification of a specific phase or the discovery of an unknown polymorph always requires indexing of the associated diffraction pattern. However, despite the importance of this procedure, only a few approaches have been developed so far. Recently, an advanced mathematical framework for indexing of these specific diffraction patterns has been developed. Here, the successful implementation of this framework in the form of an automated indexing software, named *GIDInd*, is introduced. *GIDInd* is based on the assumption of a triclinic unit cell with six lattice constants and a distinct contact plane parallel to the substrate surface. Two approaches are chosen: (i) using only diffraction peaks of the GIXD pattern and (ii) combining the GIXD pattern with a specular diffraction peak. In the first approach the six unknown lattice parameters have to be determined by a single fitting procedure, while in the second approach two successive fitting procedures are used with three unknown parameters each. The output unit cells are reduced cells according to approved crystallographic conventions. Unit-cell solutions are additionally numerically optimized. The computational toolkit is compiled in the form of a MATLAB executable and presented within a user-friendly graphical user interface. The program is demonstrated by application on two independent examples of thin organic films.

## Abstract 2

The *GIDInd* software package is a MATLAB-based application for automated indexing of grazing-incidence X-ray diffraction data.

## 1. Introduction   

The formation of potentially new polymorphs due to the transition from bulk to thin films is a well know phenomenon (Jones *et al.*, 2016 ▸; Gentili *et al.*, 2019 ▸). The presence of a substrate surface during the crystallization process can induce crystal structures with new molecular packing motifs. Frequently, the crystallization at surfaces is associated with a strong preferred orientation of the crystallites, which exhibit a well defined crystallographic plane (called the contact or texture plane) parallel to the substrate surface. In many cases, no azimuthal (*i.e.* in-plane) order of the crystallites is observed owing to the isotropic nature of the substrate surfaces. This type of crystalline orientation is called uniplanar texture (Heffelfinger & Burton, 1960 ▸) or fibre texture (Roe & Krigbaum, 1964 ▸).

Grazing-incidence X-ray diffraction (GIXD) has become a sophisticated technique for structural characterization of thin films. The primary X-ray beam with the wavevector **k**_0_ and the scattered X-ray beam with the wavevector **k** determine the scattering vector **q** by **q** = **k** − **k**_0_. According to the Laue equation, diffraction occurs if the scattering vector **q** is equal to a reciprocal lattice vector **g**. The surface sensitivity in a GIXD setup is high, making it particularly well suited for investigations of crystalline properties of thin films. Just as in single-crystal X-ray diffraction (XRD) and powder X-ray diffraction, the crystallographic unit cell is obtained by indexing of the diffraction data. While in case of single-crystal XRD usually all three components of the scattering vector are utilized for the indexing procedure (Pan *et al.*, 2012 ▸; Sauter *et al.*, 2004 ▸), powder diffraction uses only the length of the scattering vector (Boultif & Louër, 2004 ▸; Coelho, 2003 ▸). GIXD investigations provide two components of the scattering vector: the in-plane component *q_xy_*, aligned parallel to the substrate surface, and the out-of-plane component *q_z,_* perpendicular to the substrate surface. These two components are connected to the total length of the scattering vector by 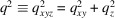.

A few approaches for processing experimental GIXD data have been developed (Smilgies & Blasini, 2007 ▸; Breiby *et al.*, 2008 ▸; Hailey *et al.*, 2014 ▸; Jiang, 2015 ▸; Schrode *et al.*, 2019 ▸; Savikhin *et al.*, 2020 ▸). Not all of them address the task of indexing with regard to unit-cell determination and even fewer consider the contact plane of the crystallites and the substrate surface as separate parameters necessary to determine. For this reason, the rotation matrix of the thin-film crystallites relative to the substrate surface must be considered (Shmueli, 2006 ▸). If the lattice parameters are, however, unknown, both the lattice constants and the rotation matrix need to be determined, which is a much more challenging task.

A significant improvement of the issue is the computational tool *Diffraction Pattern Calculator *(*DPC*) (Hailey *et al.*, 2014 ▸). It was the first toolkit that incorporated the determination of the unit-cell parameters by simultaneous assignment of the Laue indices. Prior to the unit-cell analysis, several operation parameters have to be defined by the user. Although the toolkit integrates many features, the high amount of necessary input and previous knowledge for indexing could be a limitation of the *DPC*.

A recent tool addressing the indexing problem of GIXD patterns is the MATLAB-based software package *GIWAXS-SIIRkit* (Savikhin *et al.*, 2020 ▸). Diffraction patterns can be optionally analysed to generate data sets of *q_xy_* and *q_z_*. Similarly to our presented algorithm (Simbrunner *et al.*, 2018 ▸, 2019 ▸), *GIWAXS-SIIRkit* splits the indexing process into two parts. In the first part, the *q_xy_* data are preliminarily indexed by tuples of two Laue indices (*hk*). This is achieved by evaluation of the in-plane component of the scattering vector. Solutions obtained that way are used to assign indices to the remaining peaks. The program cycles through the index permutations within a range of −8 to 8, forms the difference for every case and thereby searches for a minimum in Δ*q_xy_*. In the second indexing step, the out-of-plane components of the scattering vectors are evaluated. The result of this algorithm is the unit-cell solution with the lowest total error expressed in Cartesian distances. This routine is limited to problems where the (001) plane is the plane parallel to the substrate surface. If a contact plane with any Miller indices exists, the reduced unit cell can be linearly transformed to a supercell where the (001) plane is the new contact plane.

In our previous work, we exploited a combination of GIXD data together with information acquired by specular X-ray diffraction. In specular XRD geometry, the angle between the primary beam and sample surface is the same as the angle enclosed by the scattered beam and sample surface. The presence of a specular diffraction peak indicates that a defined crystallographic plane (contact plane) of the crystallites is oriented parallel to the substrate. If the contact plane of the crystallographic unit cell with the substrate surface is considered an additional deducible quantity, a novel mathematical treatment for the indexing procedure of the GIXD data is required. Consequently, additional unknown parameters must be determined by the algorithm. A comprehensive formalism that addresses exactly this problem is proposed in recent work (Simbrunner *et al.*, 2018 ▸, 2019 ▸, 2020 ▸). The general (triclinic) case as well as systems of higher symmetry have been dealt with.

In this article a freely available, automatic indexing software called *GIDInd* is presented, in which our algorithm has been implemented. We have included the option to search for either a monoclinic or a triclinic unit cell without prior knowledge of the specular diffraction peak from specular XRD. However, owing to the amount of computational work required, this method is much more time consuming and the inclusion of the specular scan is recommended.

## 2. Methods   

### 2.1. Indexing formalism   

With GIXD measurements, two individual components of the scattering vector **q** are accessible. Here, every point in the two-dimensional reciprocal-space map is described by a tuple of *q_xy_* and *q_z_*. These peak positions (or reflections) are the required input data for the here-proposed formalism as well as for *GIDInd*. After appropriate substitution, the potential lattice constants *a*, *b*, *c*, α, β and γ are obtained by solving sets of linear systems of equations (LSEs) under variation of the three Laue indices *h*, *k* and *l*. Using the GIXD data only (without the use of a specular diffraction peak at a defined *q_z_* value and *q_xy_* = 0), this approach is a single-step procedure with every solution yielding one potential set of lattice constants. Owing to the high number of possible combinations of the three Laue indices, this can be computationally challenging, often limited by performance and memory of the available computer resources.

A specular diffraction peak provides data in a region that is not accessible with conventional GIXD experiments. This additional peak at *q_xy_* = 0 allows splitting the determination of the lattice constants into two consecutive parts. The presence of a specular peak reveals that the orientation of the crystallographic cell on the substrate surface can be expressed by a contact plane, which is a defined net plane of the crystal denoted by Miller indices. In this way, it is not necessary to compute and evaluate an entire set of lattice parameters for every single combination of integer numbers that constitute the Laue indices of diffraction. It is rather possible to intervene at an earlier point and to apply sorting and filtering to the numerical solutions, thereby keeping the computational effort at a much lower level.

#### 2.1.1. Formalism using a specular diffraction peak   

The indexing formalism used in this first part of the work considers the contact plane [with Miller indices (*uvw*)] of the sample with respect to the substrate surface as a separate set of integer parameters. The consequence of such a defined crystallographic plane is the (possible) occurrence of a specular diffraction peak. By systematic variation of the integer numbers and additional incorporation of the position of a specular diffraction peak, the above-mentioned separation of the lattice parameter calculation becomes feasible. For the comprehensive analytical derivation of the mathematical framework we refer to earlier work (Simbrunner *et al.*, 2019 ▸, 2018 ▸). Important for this algorithm is the accessibility of the individual components of the reciprocal lattice vectors. The equations for the components are adopted without further analytic derivation and are summarized in Table 1 ▸.

For every given diffraction peak, the Laue condition must be fulfilled for the total length of the scattering vector with *q_xyz_ = g_xyz_*, and consequently for the components *q_xy_ = g_xy_* and *q_z_ = g_z_*. This must also be valid for the specular diffraction peak with *q*_spec_ = *g*_spec_. We can therefore relate every pair (*q_xy_*, *q_z_*) to a point in reciprocal space (*g_xy_*, *g_z_*), if the Laue indices and the unit-cell parameters are known. Conversely, the unit-cell parameters must be derivable for a given set of Laue indices and for a known point (*q_xy_*, *q_z_*).

**Figure 1:**
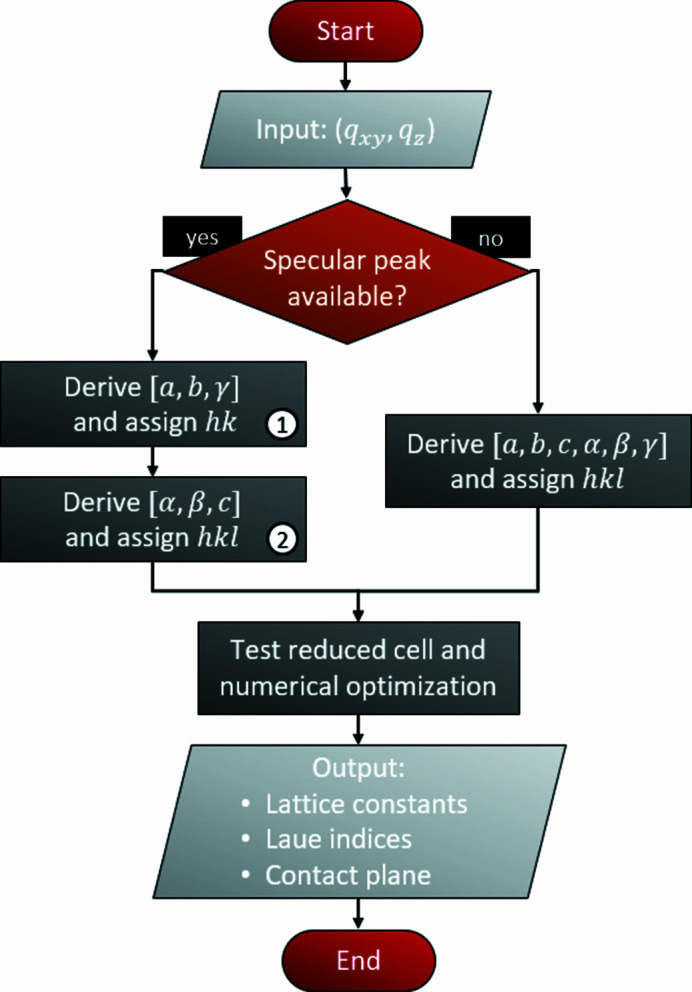
Process workflow of the indexing algorithm realized with *GIDInd*. The markers on the left branch indicate the split processing of the peak positions (*q_xy_*, *q_z_*) for derivation of the lattice parameters [*a*, *b*, γ] followed by [α, β, *c*] of the unit cell when a specular diffraction peak is available. The right branch demonstrates the single fitting procedure when working without a specular scan.

Thus, in the first part of the indexing routine, the Miller indices of the contact plane, *i.e.* the integers *u* and *v*, as well as the Laue indices *h* and *k* of the experimentally observed diffraction peaks are assigned by systematically varying the integer variables and by calculating three lattice parameters *a*, *b* and γ. In a subsequent step (i) *w* of the contact plane, (ii) the Laue indices *l* of the diffraction peaks, and (iii) the lattice constants *c*, α and β are determined. This is schematically shown in Fig. 1 ▸.

Our algorithm builds upon the following mathematical expression:
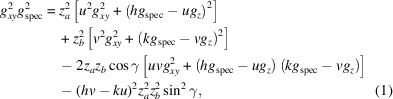
where *g*_spec_ is the magnitude of the reciprocal lattice vector associated with the specular diffraction peak, 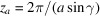 and 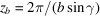. In equation (1), which is valid for any contact plane (*uvw*), the real-space parameters are reduced to *a*, *b* and γ. The unknown integers are the Miller indices *u* and *v* and the Laue indices *h* and *k* for every observed Bragg peak. Here, the first indexing step contains the determination of sets of (*u*, *v*, *a*, *b*, γ) with potential values for (*h*, *k*). We will follow this choice of order throughout this work. Equation (1) is symmetric and analogous expressions are valid for (*u*, *w*, *a*, *c*, β) with (*h*, *l*) and (*v*, *w*, *b*, *c*, α) with (*k*, *l*).

Equation (1) facilitates the mathematical analysis, where the integer variables can be varied and only three real-space unknowns have to be calculated. Therefore, when indexing GIXD patterns, the acquisition of a specular diffraction peak is of considerable help. The unknown real-space parameters *a*, *b* and γ can be calculated from the *q_xy_* and *q*_*z*_ values of three independent Bragg peak series. This can be achieved analytically by employing appropriate mathematical substitutions to obtain linearly solvable equations (see Appendix *A* [App appa]).

Following this procedure, several sub-sets with solutions of (*u*, *v*, *a*, *b*, γ) are obtained. These are used to subsequently index the measured components *q_xy_*. By computing the vector components *g_xy_* (*h_i_*, *k_i_*) [*cf.* equation (1)] and searching for the smallest absolute differences, tuples of Laue indices can be assigned to the experimental diffraction peaks. A set of (*u*, *v*, *a*, *b*, γ) is then sorted and evaluated with respect to its summed root-mean-square deviation Δ*q_xy_*. This is one of the core tasks of *GIDInd* and is elaborated in Section 3. The final solutions are reduced because of the restrictions imposed by Niggli’s scalar-product criterion (Niggli, 1928 ▸) and by optional user-selected boundary conditions on *a*, *b* and γ.

The remaining lattice constants α, β and *c* together with the third Miller index *w* of the contact plane (*uvw*) can be acquired in a similar way using the expressions for the out-of-plane component *g_z_* of the reciprocal-lattice vector and for the specular component *g*_spec_ from Table 1 ▸. More mathematical details are given in Appendix *B* [App appb].

This algorithm, when applied in a reasonable range for the Miller indices (*uvw*) of the contact plane and the Laue indices *hkl* of all observed Bragg peaks, allows us to derive numerically possible sets of parameters in the form of (*u*, *v*, *w*, *a*, *b*, *c*, α, β, γ). Using these sets, the full data (namely every tuple of *q_xy_* and *q_z_*) can be indexed and the deviations not only in *q_xy_* but also in *q_z_* and especially in the total length *q_xyz_* are evaluable. The here-proposed indexing software is used to apply these equations in a highly automated manner, thereby deriving the best-fitting lattice constants for a given set of Bragg peaks from GIXD experiments.

#### 2.1.2. Formalism without the use of a specular diffraction peak   

In the triclinic case, the following expressions for the total length of the reciprocal-lattice vector 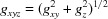 and the out-of-plane component *g_z_* (see Table 1 ▸) are used:
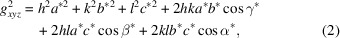
where 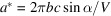, 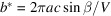, 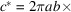 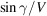, 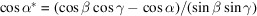, 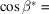 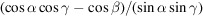 and 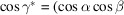 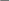 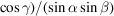 are the reciprocal cell parameters and *V* is the unit-cell volume, which can be explicitly written as
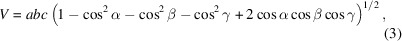
and
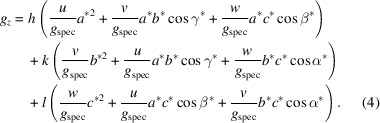
In a first step, equation (4) is built up as overdetermined LSEs by choosing the *q_z_* of four reflections and varying the Laue indices *h_i_*, *k_i_* and *l_i_* for *i* = 1–4. The solutions with the smallest residual error are further processed and expanded by adding two additional reflections and varying their Laue indices. Using equations (2) and (4), the resulting LSEs with the smallest residual errors are considered as tentative solutions. By continuously adding the *q_xyz_* and *q_z_* of further reflections and by varying the associated Laue indices, the overdetermined LSEs are expanded and the best solutions are determined. Requiring considerable computational work, this procedure is of course time consuming. Therefore, the Laue indices of the first six equations, which must be linearly independent, can only be varied in a small interval. For this reason, the reflections with the smallest *q_xyz_* are chosen first. Restrictions on the final lattice parameters are imposed by the Niggli criteria (Niggli, 1928 ▸) and by optionally chosen boundary conditions.

The orientation parameters of the final unit cell are determined by using equation (4) (see Appendix *C* [App appc]).

In the monoclinic lattice, equations (2) and (4) reduce to
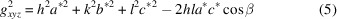
and
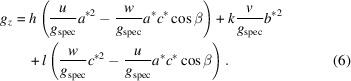
Then, of course, our algorithm results in less computational work and is less time consuming. Therefore, we offer the option – in a first attempt – to check if the data are compatible with a monoclinic system.

### 2.2. Common final steps   

In both implemented algorithms, that using the specular diffraction peak and that not using the specular diffraction peak, the final solutions for the lattice constants are optimized by performing first-order corrections (Simbrunner *et al.*, 2020 ▸). Furthermore, it is checked whether the final unit cells correspond to the reduced cells (Buerger, 1957 ▸). Redundant solutions due to the symmetry of the equations are removed.

## 3. Indexing software: *GIDInd*   

The programmatic implementation of the algorithm is based on MATLAB and provided as a standalone desktop application. The peak positions given in the form of positive tuples of (*q_xy_*, *q_z_*) are the required input for the program. A proven and efficient tool to derive the diffraction peaks from experimental GIXD data is the software package *GIDVis* (Schrode *et al.*, 2019 ▸). For *GIDInd*, the input data have to be provided within a formatted .xls (or .xlsx) file. Each peak position is listed in a row with numerical values of *q_xy_* in the first column and the corresponding values for the out-of-plane components *q_z_* in the second column. If applicable, the specular peak must be included directly in the uploaded file in the row where *q_xy_* = 0. The uploaded file should contain only numeric floating-point numbers with a dot for decimal separation. If the formatting is accepted and at least one specular diffraction peak is included, the data are added to the *q* map within the graphical user interface (GUI). Multiple indication icons and status messages are provided to guide the operator through the indexing process. If no specular information is available within the list, a subroutine is enabled addressing this case.

### 3.1. Indexing using a specular diffraction peak   

**Figure 2:**
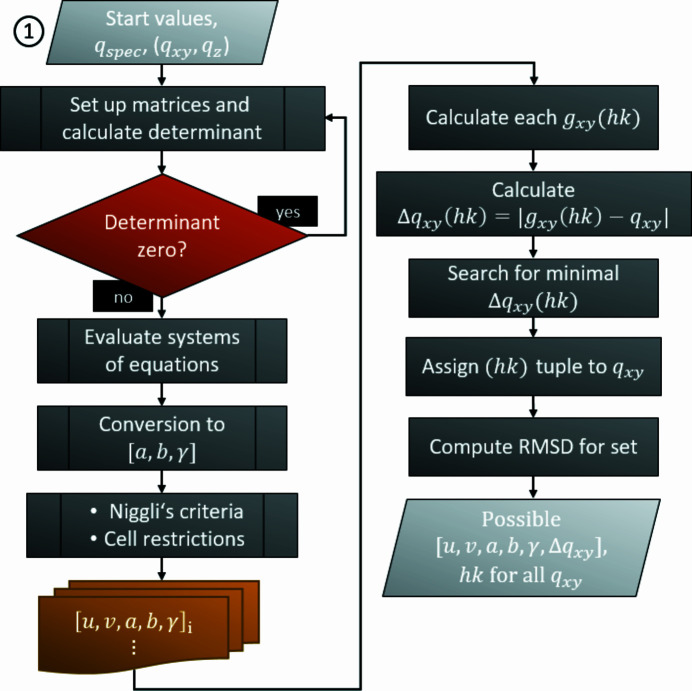
Process diagram describing the determination of the lattice parameters [*a*, *b*, γ] using the diffraction peaks (*q_xy_*, *q_z_*) and the specular peak *q*_spec_. After comparison with calculated values for the component of the reciprocal-lattice vector *g_xy_* (*hk*), the whole branch results in sub-sets containing the three lattice parameters, preliminarily assigned pairs of Laue indices *h* and *k*, two Miller indices *u* and *v*, and an RMSD for the set expressed as *Δq_xy_*.

The indexing procedure and the calculation of the real-space lattice parameters is split into two consecutive parts if the specular peak is utilized (see Section 2.1.1). The internal architecture of the first part, namely derivation of *a*, *b* and γ upon successive assignment of the Laue indices *h* and *k* to *q_xy_* data, is outlined in Fig. 2 ▸. For setting up the LSEs [equation (12), Appendix *A*], ‘start values’ are specified by the routine. These are linearly independent sets of three Bragg peaks from the provided reciprocal-space map. That has a direct impact on the input file containing the Bragg peaks: for indexing GIXD data with *GIDInd* using a specular diffraction peak, at least four independent peaks, with one line having the entry *q_xy_* = 0, are required. The approach elaborated in Appendix *D* [App appd] has been shown to be successful in checking the (in)dependence. After these initialization steps, the matrices for the LSEs are constructed. For every combination of integers (*u*, *v*, *h*, *k*) in a specified range, a quadratic array is set up. The pre-adjusted limit for permuting the Miller indices is ±2. The option to restrict the solutions to a (001) contact plane or to any other desired combination of fixed (*uvw*) is also provided to the operator. The default limit for *h* and *k* is ±3, but this is adjustable if required. This choice is reasonable, as the ‘start sets’ to compute *a*, *b* and γ are generated using the lowest *q* values first, and the Laue indices are typically the lowest in this region. This assumption is in agreement with other reported programs addressing the indexing problem of GIXD patterns (Savikhin *et al.*, 2020 ▸; Hailey *et al.*, 2014 ▸). The evaluation of every LSE is based on MATLAB’s symbolic matrix left division for every matrix with a determinant unequal to zero. The substitutions given in Appendix *A* [App appa] are applied to derive the sub-sets with solutions of (*u*, *v*, *a*, *b*, γ). For machines with available multicore processors, these calculations are executed in parallel computing algorithms using parallelized loops.

Crystallographic restrictions, formulated as Niggli criteria (Niggli, 1928 ▸), as well as user-defined limitations to the lattice constants allow a first confinement of the possible solutions at this point. Non-real or negative numerical results are eliminated and only solutions with 60 ≤ γ ≤ 120° (Hahn, 2005 ▸) are considered for the subsequent indexing procedure. Additionally, the lattice constants *a* and *b* are restricted to the range between 3 and 60 Å. Adjustment of an upper limit at ∼30 Å is recommended, to keep computational effort low.

During this part of the routine, every experimental peak position gets preliminarily equipped with four potential pairs of *h* and *k*. (An unambiguous assignment of the Laue indices is not possible at this point owing to symmetry considerations of the equations provided.) The lower and upper limits are set to ±6 by default and adjustable to a maximum of ±8. This program parameter can be differentiated from the maximum cap for *h* and *k* for solving the LSEs, as not only the lowest values out of the *q* map are considered now, but all input diffraction peaks.

**Figure 3:**
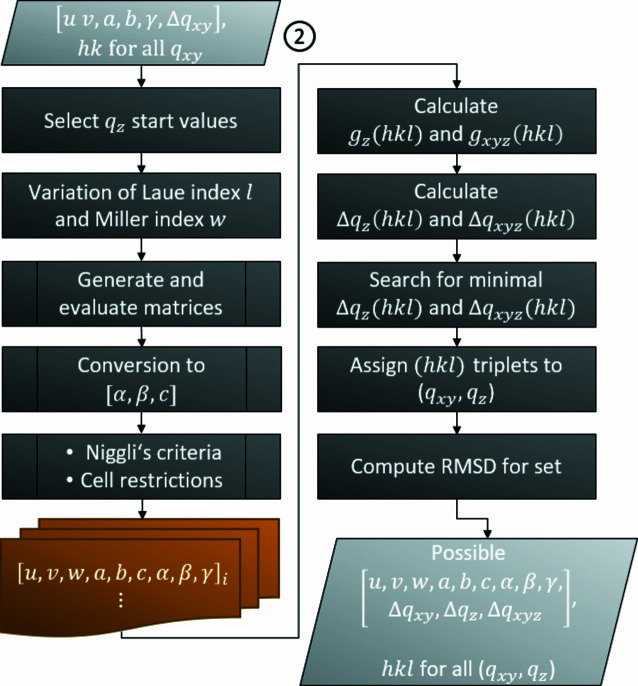
Process diagram containing the branch for derivation of the preliminary lattice constants with the associated RMSDs *Δq_xy_, Δq_z_* and* Δq_xyz_* before reduction and optimization is applied. In addition to the first step, the out-of-plane component *g_z_* (*hkl*) and the total length of the reciprocal-lattice vector *g_xyz_* (*hkl*) are used for assignment of the Laue indices *h*, *k* and *l*.

For a given sub-set of (*u*, *v*, *a*, *b*, γ), the program computes *g_xy_* (*hk*) for every single combination of *hk*. The four tuples of *hk* yielding the smallest absolute differences *Δq_xy_* (*hk*) are assigned to a chosen value of *q_xy_*. This is done for each of the *N* input Bragg peaks and the ‘quality’ of the set is derived by computing the summed root-mean-square deviations (RMSD) with
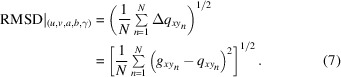
This algorithm applied to all possible sub-sets will result in two outputs: (i) a list of diffraction peaks with assigned pairs of potential Laue tuples and (ii) the corresponding list of partial solutions (*u*, *v*, *a*, *b*, γ). This serves as input for the second part of the indexing routine. As demonstrated in Fig. 3 ▸, the derivation of the remaining parameters follows a similar process architecture. In a first step, ‘start values’ out of the *q_z_* data are selected. For generation of the LSEs [equation (22), Appendix *B* [App appb]], the three lowest values which are not multiples of each other are considered. The limit for variation of the Laue index *l* is a program parameter and thus adjustable by the user. By default, *l* is varied in the range between −6 and +6 and adjustable to a maximum of ±8. If not specifically defined through a GUI input, the cap of *w* is internally adopted to the maximal occurring value of *u* and *v*.

The program generates one LSE for every possible numerical combination. The number of systems to be solved is therefore directly dependent on the range of the indices and on how many previously acquired solutions (sub-sets) are included in the second round. This can also be controlled from the GUI. The so-obtained LSEs can again be solved using MATLAB’s matrix left division function, which returns the least-squares solution to each overdetermined system of equations. The norm of the residuals is used as a sorting quantity at this point. Using the previously defined substitutions (see Appendix *B* [App appb]), the three remaining cell parameters are deduced for each system. The routine presents the resulting sets in the form of (*u*, *v*, *w*, *a*, *b*, *c*, α, β, γ) together with the list of peak positions and the tentatively assigned tuples of *h* and *k*. Again, the (purely numerical) output of lattice constants can be gradually confined and reduced by the application of user restrictions and by continuous inquiries regarding the fulfillment of Niggli’s criteria. Only those sets passing these tests will continue to be considered for full indexing in the second part.

During the first indexing step, every Bragg peak is equipped only with preliminarily assigned pairs of Laue indices *h* and *k*. At this point of the indexing routine, the third Laue index *l* remains to be determined for every single reflection. This could be done by again varying integer numbers in a certain range and evaluating every single possible combination regarding the overall deviation of the diffraction pattern. However, the equations from Table 1 ▸ allow the value for *l* to be determined analytically, as all other formerly unknown parameters are now available. This option is preferred to minimize computational time and enhance the overall performance of the program. For a fixed set of (*u*, *v*, *w*, *a*, *b*, *c*, α, β, γ), the value of *l* is acquired for each reflection and each combination of *h_i_*, *k_i_* where *i* = 1–4. It is subsequently rounded to the next integer number. By applying exactly the same equations again, every value for *g_z_* (*hkl*) and *g_xyz_* (*hkl*) can be computed. The program searches for the smallest absolute differences Δ*q_z_* (*hkl*) and Δ*q_xyz_* (*hkl*) for every combination. This time, the triplet *hkl* with the smallest deviation in *g_xyz_* is assigned to the reflection and settled as the final set of Laue indices. As already introduced in equation (7), the ‘quality’ of the as-derived individual solutions is again assessed using the summed root-mean-square deviation.

With all Laue indices derived for all provided Bragg peaks, each single set of lattice parameters undergoes a numerical optimization routine based on first-order corrections (Simbrunner *et al.*, 2020 ▸). This subroutine computes correction terms for the six lattice constants *a*, *b*, *c*, α, β and γ in order to minimize the summed RMSDs in the component *q_z_* and in the total length *q_xyz_* of the scattering vectors. Even though the received solutions face minimal numerical errors with regard to the experimental diffraction pattern, these unit-cell solutions are not necessarily unique and superlattices can appear. Each solution is therefore checked to see if it corresponds to the reduced cell (the unit cell based on the three shortest non-coplanar lattice vectors) and, if not, adjusted accordingly. If possible, similar and reoccurring results are combined by comparison of their Laue indices. Those exhibiting the smallest errors are presented in an ascending order to the user through the GUI of *GIDInd*. The results are provided to the operator in the form of numbered lists with the six lattice parameters *a*, *b*, *c*, α, β and γ, the three Miller indices of the contact plane *u*, *v* and *w*, the volume *V* of the cell, and the four summed errors in *q_xy_*, *q_z_*, *q_xyz_* and *q*_spec_. Using the GUI, the simulated diffraction patterns for every solution can be calculated to be compared with the experimental input data. The *GIDInd* interface offers options to generate compiled output files (.xlsx format) with all information of a specific solution and with all Laue indices for the corresponding Bragg peaks. The basic functionality of the GUI will be demonstrated with two samples in Section 4. For a detailed explanation we refer the reader to the user’s manual, available with the links provided in Section 5.

### 3.2. Indexing without using a specular diffraction peak   

If no specular peak is included, a subprogram is enabled. The interface of the subroutine offers several adjustments for the indexing procedure. These possible adjustments are of particular interest in this case, as the computation time and the use of memory are the liming factors when working without a specular diffraction peak. In particular, the possibility to restrict the potential Laue indices is crucial in order to make the derivation of solutions possible. The cap of the Laue indices *h* and *k* for building the LSEs is ±1 and ±2, respectively. The third index *l* is varied between −2 and +2. The subprogram is able to assign integers in the range between −6 and +6 upon subsequent indexing. An additional computational reduction of effort when deriving a solution with this routine is the possible choice between a monoclinic cell and a triclinic cell. As already implied by the indexing formalism in Section 2.1.2, the assumption of a monoclinic unit cell facilitates the mathematical treatment. Using the triclinic approach, the six lattice constants *a*, *b*, *c*, α, β and γ are derived simultaneously. The minimum number of peak positions is therefore also raised to six. In addition, these peaks must be linearly independent, which is not necessarily the case when deriving data from GIXD patterns. In the monoclinic approach, only four independent peak positions have to be provided. Note that the indexing routine without incorporation of the specular scan has no automation for checking the (in)dependence of the Bragg peaks. The use of the subroutine is demonstrated in Section 4.1.

## 4. Application examples   

In addition to the calculation of lattice parameters, different, although related, tools are provided when working with *GIDInd.* The main window consists of five sub-windows (panels): ‘Data Points and Representation Panel’, ‘Add Crystal Panel’, ‘Indexing Panel’, ‘Error Panel’ and ‘Results Panel’. For indexing without using a specular diffraction peak, a subprogram is opened for controlling the indexing procedure. The functionality of the indexing program is demonstrated here on two different thin-film samples. In both cases, the peak positions were extracted from GIXD maps using the GIXD analysis tool *GIDVis* (Schrode *et al.*, 2019 ▸).

### 4.1. Pentacene­quinone on highly oriented pyrolytic graphite   

6,13-Pentacene­quinone (PQ) is an example of an organic semiconductor material with the chemical formula C_22_H_12_O_2_. GIXD data of a PQ thin-film sample, grown on highly oriented pyrolytic graphite, are chosen to demonstrate the applicability of *GIDInd*. A specular diffraction peak is provided at *q_z_* = 1.9460 Å^−1^. In total, a set with 74 Bragg reflections is provided, of which 30 peak positions are taken for the calculation of the lattice parameters. Upon indexing of this data set, the program is applied with initial settings only, to emphasize the fast and easy-to-use approach.

**Figure 4:**
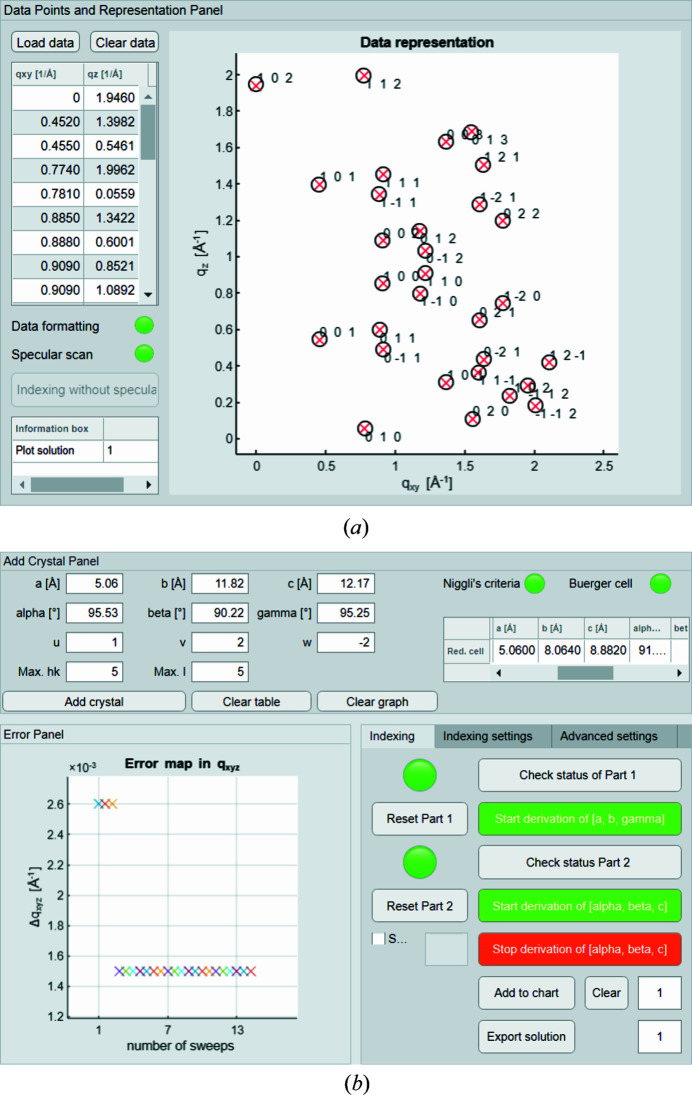
Selected screenshots of the graphical user interface of *GIDInd* after indexing. (*a*) ‘Data Points and Representation Panel’ with a selection of 30 peak positions for indexing of the PQ data set (plotted in black). The pattern printed in red represents the simulated diffraction pattern using the best solution with respect to the error in *q_xyz_*. (*b*) Cut-out section of the interface containing the ‘Add Crystal Panel’, the ‘Error Panel’ and the main control panel labelled ‘Indexing’.

The data are uploaded via the ‘Data Points and Representation Panel’ and, as the formatting is accepted and the specular diffraction peak is included within the set, the routine adds the peak positions to the ‘Data representation’ graph right away. A screenshot of the panel is shown in Fig. 4 ▸(*a*). Black markers are used to indicate (uploaded) experimental peaks and diffraction patterns simulated with the derived unit-cell parameters are printed with red cross markers. The GUI guides the operator through the indexing procedure via dialogue windows, indication icons and continually updated process bars. After successful execution of the first indexing part, the user gets possible results presented within a panel at the bottom of the GUI. The routine allows sorting of the obtained sets of (*u*, *v*, *a*, *b*, γ) either according to their summed RMSDs (*Δq_xy_*) or by the areas of the parallelograms formed through *a*, *b* and γ. Both options can be useful; however, a general recommendation for sorting the sub-sets cannot be given. It is usually expedient to search for the smallest cells with a reasonable deviation. A rough indication for a good match is *Δq_xy_* ≃ 0.01 Å^−1^ and below, but this error varies from sample to sample and depends also on the quality of the provided diffraction data. Using the indexing routine several times will certainly help the user to develop intuition about deviation values yielding the highest matching quality. For the presented example of PQ, the sets are sorted by ascending parallelogram areas, as the smallest appearing areas also exhibit the least deviations in *q_xy_*. A possible strategy for the second indexing part is monitoring the quality of the derived unit-cell solutions using the ‘Error map’, shown in the screenshot in Fig. 4 ▸(*b*). The user can stop the program manually when the summed RMSD in the total length of the scattering vector (*Δq_xyz_*) converges to a minimum. As long as the operator does not intervene, the program continues processing the earlier derived sub-sets. A defined range for the sub-sets that should be considered can be specified via the GUI. We emphasize again that the mathematical solutions are not unique. Various unit-cell solutions may be able to reproduce a matching diffraction pattern.

Here, the program is ended after nine different sets of (*u*, *v*, *a*, *b*, γ) are processed, as the error converged to a minimum value of *Δq_xyz_* = 0.0015 Å^−1^. The unit-cell solution exhibiting the least deviations with *Δq_xy_* = 0.0010 Å^−1^ and *Δq_z_* = 0.0022 Å^−1^ is given explicitly with *a* = 5.056, *b* = 8.076, *c* = 8.871 Å, α = 91.54, β = 93.03, γ = 94.14° and a volume of *V* = 360.8 Å^3^. The derived Miller indices of the contact plane are (*uvw*) = (102). This solution agrees with the recently reported new polymorph of the PQ crystal (Simbrunner *et al.*, 2018 ▸) within the expected range of uncertainty.

**Figure 5:**
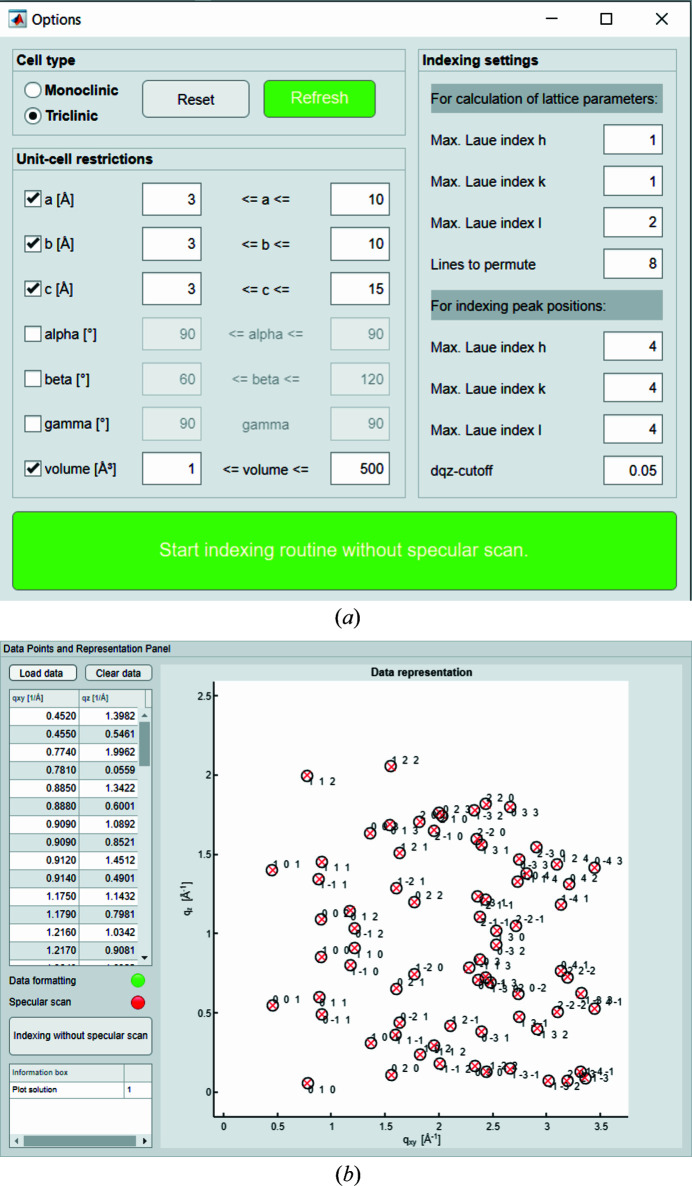
(*a*) ‘Options’ panel of the sub-routine ‘Indexing without specular scan’. (*b*) ‘Data Points and Representation Panel’ showing the simulated diffraction pattern (red) of a unit-cell solution derived with 74 input peak positions (black) without specular peak.

The same GIXD data are used for verifying the indexing algorithm without incorporating the specular diffraction peak. When uploading a file without a specular peak listed, the subroutine ‘Indexing without specular scan’ is unlocked. A screenshot of the control panel is shown in Fig. 5 ▸(*a*). The number of available and independent peaks is not always sufficient for the triclinic approach (at least six independent Bragg reflections are required). Therefore, the user can choose between the cell types ‘monoclinic’ and ‘triclinic’. As in the case where the specular peak is used, the caps for the Laue indices can be set separately. Restrictions on the real-space lattice constants and the volume are also provided to filter the output solutions. Once this sub-routine starts, it processes the input in a single fitting procedure to derive sets of (*a*, *b*, *c*, α, β, γ). The results, if available, are embedded in the GUI and can then be compared graphically or exported as mentioned above. With the here-provided 74 reflections the following unit cell results: *a* = 5.053, *b* = 8.076, *c* = 8.8671 Å, α = 91.55, β = 93.08, γ = 94.15°, *V* = 360.0 Å^3^ with *Δq_xyz_* = 0.0028 Å^−1^, *Δq_xy_* = 0.0028 Å^−1^ and *Δq_z_* = 0.0017 Å^−1^. Within the range of uncertainty, this is the same result as derived above. The corresponding simulated diffraction pattern is shown in Fig. 5 ▸(*b*). With this framework, a distinct contact plane cannot be specified with integer Miller indices. The rotation angles ψ and ϕ to calculate the rotation matrix (as described in Appendix *C* [App appc]) are stored in the generated output file. Although the results appear to be the same in the case of PQ, we report significant drawbacks in high memory occupation and time consumption if the routine is used without a specular diffraction peak. However, this application can be of advantage for the case that no specular scan is available.

### 4.2. Acetyl­salicylic acid on thermally oxidized Si(100)   

**Figure 6:**
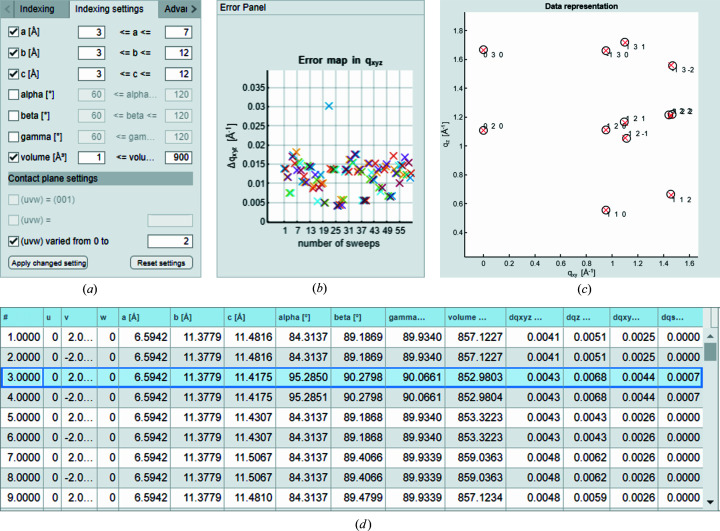
Selection of screenshots of the *GIDInd* interface. (*a*) ‘Indexing settings’ panel showing the restrictions on the potential lattice constants *a*, *b* and γ and on the volume of the crystallographic cell. (*b*) ‘Error panel’ monitoring the deviation in *q_xyz_* during execution of the indexing routine. (*c*) Input peak positions (black) of an ASS thin film together with the simulated diffraction peaks (red) calculated with the solution highlighted in the ‘Result Panel’ in (*d*).

If the initial settings (as used earlier for PQ) do not lead to sufficient solutions, a different approach for the derivation of the unit-cell parameters can be helpful. For demonstration, GIXD data of an acetyl­salicylic acid (ASS) thin film on Si(100) are evaluated. We derived ten diffraction peaks from the reciprocal-space map and, additionally, a specular diffraction peak is used for the indexing procedure with *GIDInd.* First, a run with initial settings is performed to find the tendencies according to which the lattice parameters converge and to define reasonable ranges of the errors. After this survey run, the edge lengths of the unit cell *a*, *b* and *c* and especially the volume show a clear trend towards particular values. We therefore use the ‘Indexing settings’ tab of *GIDInd* to restrict certain lattice parameters for the solution sets, as shown in Fig. 6 ▸(*a*). The screenshot of the ‘Error panel’ in Fig. 6 ▸(*b*) was taken during the survey run and shows a population of solutions around *Δq_xyz_* = 0.005 Å^−1^ and below. With the applied restrictions, we can immediately identify a unit cell which generates a matching, and therefore promising, diffraction pattern [Fig. 6 ▸(*c*)]. The corresponding unit-cell solution is shown and highlighted in Fig. 6 ▸(*d*) and explicitly given with the parameters *a* = 6.594, *b* = 11.378, *c* = 11.418 Å, α = 95.29, β = 90.28 and γ = 90.07°. The volume of the cell is *V* = 852.98 Å^3^, the total-length deviation is *Δq_xyz_* = 0.0043 and we find the plane (*uvw*) = (020) to be parallel to the substrate. The solution found here agrees with the known polymorph of ASS at ambient temperature (Wheatley, 1964 ▸; Kim *et al.*, 1985 ▸). Note again that Niggli’s criteria are implemented and the output solutions are reduced cells. The order of listing the parameters can therefore deviate from that stated in the literature. The equivalence of the obtained solutions can easily be proven using known symmetry relations (Simbrunner *et al.*, 2018 ▸).

With the possibility of restricting the parameters, the user can specifically scan regions and use potential initial guesses if available. In addition to restricting the parameters, the Miller indices can be set to pre-defined values. In some cases, it can be useful to first search for solutions that exhibit a (001) lattice plane. This can give a first impression of the possible solutions and, overall, help to save time when indexing the GIXD data with *GIDInd*.

Application of the indexing routine and the accompanying memory usage did not lead to any computational restrictions for the investigated samples. If the program parameters are kept in the ranges as demonstrated, the program should not face any programmatic problems. Nevertheless, no claim is made to completeness. Neither should the impression arise that the program has no limitations or bottlenecks, nor that the obtained solutions are unambiguous.

## 5. Availability   

The program is provided in the form of an executable file (.exe) and as a MATLAB application (.mlapp file), written and tested in MATLAB version R2019b, Update 5, on Windows OS. It is released under the terms of the GNU General Public License, either version 3 of the licence or any later version. The software can be used as a standalone executable file (MATLAB Runtime required) and as a .mlapp file (valid MATLAB licence required) together with the functions provided in the zip-compiled folder, available on the web sites stated below. The executable can be used right away with the MATLAB Runtime installed, which can be downloaded from The Mathworks Inc. (https://mathworks.com/products/compiler/matlab-runtime.html) free of charge. For individual adaptions and potential further development, all source codes are provided. To access the program, codes and further instructions, visit https://www.if.tugraz.at/amd/GIDInd/ and https://github.com/m-kainz/GIDInd. More program details, further tutorials, and additional help regarding operation and use of *GIDInd* can be found in a separate documentation file, available on the web sites.

## Figures and Tables

**Table 1:** Summary of the used equations for total length of the reciprocal lattice vectors *g_xyz_*, the magnitude of the reciprocal lattice vector associated with the specular diffraction peak *g*_spec_, and the in-plane component *g_xy_* and out-of-plane component *g_z_* of the reciprocal lattice vector **g** using the Laue indices *hkl* and the Miller indices (*uvw)* of the contact plane together with the lattice constants of the direct lattice (*a*, *b*, *c*, α, β, γ) and the reciprocal lattice parameters (*a* ^*^, *b* ^*^, *c* ^*^, α^*^, β^*^, γ^*^)

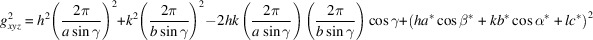
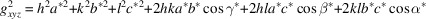
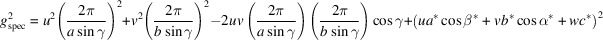
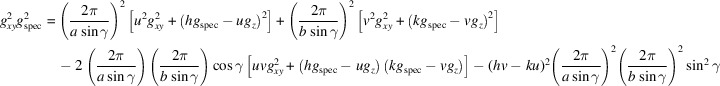
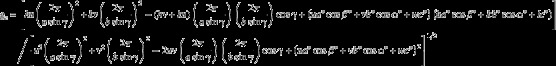
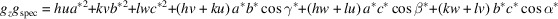

## Appendix A Mathematical procedure for analytically determining the cell parameters *a*, *b *and γ

For analytically determining the unit-cell parameters *a*, *b* and γ, it is convenient to introduce the parameters ,  and  with the substitutional relations

Note that  and  are always positive. Using these substitutions, equation (1) can be linearized and rewritten as

The parameters ,  and  can be determined from three independent Bragg peak series by solving the following LSE:

where

and *i* = 1, 2 and 3. For obtaining *a*, *b* and  from equations (8)–(10) [Disp-formula fd10] the following identity is helpful:

From equations (8)–(10) [Disp-formula fd10] and (16), the following expressions can be derived:

and

## Appendix B Mathematical procedure for analytically determining the cell parameters *c*, α and β

For setting up the LSE, the expressions for *g*_*z*_ and *g*_spec_ in Table 1 ▸ can be transformed to:

for three linearly independent peaks with indices *i =* 1, 2 and 3, and

Then, it is again possible to build an LSE of the form

Under variation of the Miller index *w* and the Laue indices *l*_1_, *l*_2_ and *l*_3_, the terms for *g*_spec,T_ and *g*_*z*,T_*i*__ have to be computed for every previously derived set of (*u*, *v*, *a*, *b*, γ). These overdetermined systems are solvable for κ, λ and ρ. The parts containing the unknown reciprocal lattice constants , , ,  and  are fully replaced by ,  and ,  − , , and  (Truger *et al.*, 2016 ▸). Then, the remaining lattice parameters can be calculated by using the following expressions:

and

## Appendix C Determining the parameters of the rotation matrix

The orientation parameters of the final unit cell are determined by using equation (4), which can be alternatively written as :

where ,  and . By building the ratios of these factors, the Miller indices of the contact plane may be found.

The rotation matrix **R** can be expressed as

where ψ and ϕ are the rotation angles. Then the following relations are valid (Simbrunner *et al.*, 2018 ▸):

For ϕ, the following relations can also be derived (Simbrunner *et al.*, 2021 ▸):

where

and

where

If *u_q_* = *v_q_* = 0, the contact plane is (001) (unrotated system).

## Appendix D Determining the start values for the linear systems of equations

To assess whether or not numerical values are multiples of one another, the following algorithm is used: For various pairs out of *N*, the total number of input Bragg peaks, the quotient of two different points  and  is computed, where  and . If the absolute difference between the quotient and its nearest integer neighbour is below a certain numerical limit (*e.g.* ε_c_ = 0.1), the two values are considered as linearly dependent. A pair  and  where

are considered to be numerically independent and a triplet of such independent values is referred to here as a ‘start set’. The routine switches through a specified number of *q_xy_* values, permutes the lines in every possible way and thus creates a series of different sets. Initially, these sets are chosen independently from any user-defined numerical limit. The number of points included, however, can be adopted via the user interface. If there is at least one possible, independent combination of three Bragg peaks, it can be found with the algorithm.
